# Culturally integrated Mandarin instruction and intercultural communicative competence: the mediating role of intercultural willingness to communicate

**DOI:** 10.3389/fpsyg.2026.1929538

**Published:** 2026-10-26

**Authors:** Chanchan Ding

**Affiliations:** College of International Education and Social Development, Zhejiang Normal University, Jinhua, China

**Keywords:** Chinese language learning, culturally integrated instruction, educational psychology, intercultural communicative competence, intercultural willingness to communicate, Mandarin as a second language, quasi-experimental design

## Abstract

**Introduction:**

Intercultural communicative competence (ICC) has become a target outcome of second and foreign language education, yet evidence on how culturally integrated instruction develops this competence in Mandarin learners, and through what psychological pathway, remains thin. This study tested whether culturally integrated Mandarin instruction—teaching that builds cultural comparison, small-group discussion, role-play, misunderstanding-repair tasks, and short reflections into communicative tasks, so that culture becomes the material of language work rather than a supplementary lecture—improves learners’ overall ICC and overall intercultural willingness to communicate (IWTC), and whether IWTC accounts for the link between instruction and competence.

**Methods:**

Using a quasi-experimental pre-test/post-test control-group design, Mandarin-as-a-second-language learners were assigned to an experimental group receiving an 8-week, weekly 90-min culturally integrated program or to a control group receiving regular Mandarin instruction. The main analysis retained 62 learners who completed both testing points and attended at least 75% of sessions (experimental *n* = 31; control *n* = 31). ICC and IWTC were measured with self-report scales scored on total-scale values.

**Results:**

The experimental group improved on ICC (pre 2.958 to post 3.650) and IWTC (pre 2.859 to post 3.519), while the control group remained essentially unchanged. After adjustment for baseline scores, group membership was associated with higher post-test ICC (*p* = 0.005) and post-test IWTC (*p* < 0.001). A bootstrap mediation model indicated that IWTC largely accounted for the association between instruction and ICC, with a significant indirect effect (0.520, 95% CI [0.256, 0.823]) and a non-significant direct effect.

**Discussion:**

Because the mediator and outcome were measured concurrently, the results are consistent with—rather than demonstrate—that culturally integrated Mandarin instruction supports competence gains by increasing learners’ readiness to engage in intercultural communication.

## 1 Introduction

Global mobility and routine cross-language contact have reshaped what counts as competence in second and foreign language education. Interpreting meaning across cultural boundaries, sustaining interaction under uncertainty, and adjusting one’s own behavior have become recognized educational outcomes (Barili and Byram, 2021; Deardorff, 2006). Intercultural communicative competence names this capacity, and classroom research shows it can be taught and assessed as a developmental outcome, not merely absorbed as a by-product of exposure (Huang, 2021; Lee et al., 2023). On this account, what learners need extends beyond grammar, vocabulary, and fluency to include the disposition and skill to negotiate meaning with people who do not share their assumptions. The salience of this need is increasingly evident from the learners’ own side: in internationalized higher-education settings, intercultural communicative competence has been shown to shape the quality of student-to-student interaction, underscoring learners’ stake in developing it, and learners increasingly look to their institutions to build this capacity as part of their own preparation for study and work across cultures (D'Orazzi and Marangell, 2025).

International Chinese language education raises the same demand in a specific form, and does so as Mandarin continues to gain international reach and is studied well beyond heritage communities, sharpening the need to understand how these learners develop intercultural competence rather than linguistic form alone. Mandarin learners work with a language whose everyday use is saturated with culturally patterned meaning in address forms, indirectness, politeness, and hospitality, so communicative success depends on reading cultural context, not just decoding forms (Liu, 2022; Lv et al., 2021). Studies of Mandarin learners and Chinese-as-a-second-language settings have started to document how competence develops in these conditions (Dai et al., 2025; Gong et al., 2022), and work on Chinese learners of additional languages signals broader growth in intercultural research in Chinese educational contexts (Gui, 2024). The pattern across these strands is consistent: culture in the Mandarin classroom is too often treated as background information and rarely used to support communicative development.

The available research base is informative but uneven. Much of it focuses on teacher cognition, curricular description, or pragmatic and communicative tendencies of learners observed correlationally (Dai et al., 2025; Gong et al., 2022; Li, 2025; Liu, 2022; Lv et al., 2021). These contributions clarify how practitioners conceive of intercultural teaching and how learner attributes cluster, yet they were not designed to test whether a defined instructional intervention moves learner competence. Teacher-level insight, however valuable, does not stand in for learner-level effect.

Three limitations follow. Learners of Mandarin remain under-represented as the unit of analysis in intervention research, so claims about culturally integrated teaching often rest on activity descriptions, with little controlled comparison (Lee et al., 2023; Wibowo et al., 2024). Researchers have rarely modeled the mechanism —why such teaching would raise competence—even though intercultural willingness to communicate is treated elsewhere as a meaningful predictor of communicative behavior and competence (Fathi et al., 2023; Wang and Cionea, 2024). And where technology-rich or immersive interventions have shown promise for competence and communicative readiness, the underlying pathway has usually been left implicit (Fathi et al., 2025). What is conspicuously missing is learner-level intervention evidence that specifies both an instructional effect and its psychological mechanism in a Mandarin-learning context; despite the practical importance of the field, controlled tests that model why culturally integrated teaching would raise competence remain largely absent.

This study addresses that gap with a quasi-experimental test of culturally integrated Mandarin instruction and a mediation model that positions intercultural willingness to communicate as the psychological route from instruction to competence (Choi, 2024; Fathi et al., 2023; MacIntyre et al., 1998; Wang and Cionea, 2024). Three questions organize the work. Does culturally integrated Mandarin instruction improve learners’ overall intercultural communicative competence relative to regular instruction? Does it enhance learners’ overall intercultural willingness to communicate? Does intercultural willingness to communicate mediate the relationship between instruction and competence?

## 2 Literature review and hypothesis development

### 2.1 Intercultural communicative competence in second language education

Intercultural communicative competence refers to the ability to understand, interact, and communicate effectively and appropriately across cultural boundaries—a bundle of knowledge, attitudes, skills, awareness, and behavioral orientation rather than a store of cultural facts (Barili and Byram, 2021; Deardorff, 2006). A learner who can recite customs but freezes in an unfamiliar exchange has cultural information, not competence; competence shows in interpretation, adjustment, and sustained engagement. Framing the construct this way keeps cultural awareness, only one component, from being mistaken for the integrated, performable capacity that competence names.

Intercultural communication itself spans two interwoven dimensions: intercultural verbal communication (IVC), which carries meaning through language, and intercultural non-verbal communication (INC), which works through gesture, gaze, proxemics, and the other channels that accompany talk. Byram (2021) revisits the role of non-verbal communication in intercultural interaction but stops short of integrating it into the teaching and assessment of the construct. Because the two dimensions co-occur in interaction, teachers and learners construct their verbal and non-verbal intercultural identities together in multilingual, multicultural classrooms (Yang, 2018), and intercultural non-verbal communication competence (INCC) can matter as much as—or in some settings more than—intercultural verbal communication competence (IVCC), so it warrants deliberate development alongside it (Yang, 2025). The present study centers on the verbal dimension (IVC and IVCC) and does not separately assess non-verbal competence, which it treats as a complementary dimension of the broader construct left to future work.

This competence develops in language classrooms not automatically, but in response to designed experience. Task-based work, engagement with cultural incidents, virtual and project-based exchange, and structured reflection have each been linked to competence growth in foreign language settings (Huang, 2021; Lee et al., 2023; Liao, 2024; Reader, 2024). For instance, Huang's (2021) study of EFL learners in Taiwan tied classroom cultural work to measurable competence, and Liao's (2024) use of critical incidents with advanced learners points to interpretation and reframing—not information transfer—as the developmental engine. Much of this evidence comes from English-medium contexts, leaving open how far the same instructional logic extends to Mandarin.

A recurring thread connects competence to communicative readiness. In correlational work, learners more disposed to enter intercultural interaction show higher intercultural competence, and apprehension and perceived competence both shape whether learners are willing to communicate across cultures (Fathi et al., 2023; Miauw and Guo, 2021; Wang and Cionea, 2024). This association is suggestive, not settled: it identifies a plausible link between willingness and competence without establishing that willingness carries an instructional effect. The present model addresses the gap between a documented correlation and an untested mechanism.

### 2.2 Culturally integrated Mandarin instruction

Culturally integrated Mandarin instruction, as used here, denotes teaching that builds cultural comparison, small-group discussion, role-play, misunderstanding-repair tasks, and short reflections into the core of language work, so that culture becomes the material of communicative tasks rather than a supplementary lecture (Dai et al., 2025; Gong et al., 2022; Liu, 2022). The defining move is integration: cultural content becomes something learners compare, enact, negotiate, and reflect on. This approach separates itself from both ordinary language drilling and stand-alone cultural briefings that leave the interactional demand untouched.

The design logic here draws on task-based language teaching, in which learners work toward a communicative goal that requires real language use, with attention to meaning taking precedence over the practice of isolated forms, and with the completion of the task—not the rehearsal of a structure—organizing the lesson (Ellis, 2003). Cultural integration extends this logic by making cultural interpretation and negotiation the substance of the task itself. The resulting activity repertoire is concrete and reproducible. Liu's (2022) account of intercultural activities in Mandarin teaching for international students in China describes translation and comparison, situation setting, and role-play as workable classroom formats, and virtual-exchange and project-based designs offer additional task templates that place learners in genuine communicative positions (Peraza and Furumura, 2022; Yang and Samat, 2023). These formats share a shift in cognitive load toward the learner, who must produce, interpret, and repair meaning in a culturally loaded scenario, not merely hear how politeness or indirectness works.

A critical qualification shapes how such instruction should be designed. Approaches that teach culture as a set of fixed, group-level traits risk an essentialist framing, in which learners come away with generalized images of “how Chinese people behave” rather than an ability to read situated, variable, and often contested cultural meaning; explicit cultural content in the language classroom can, if handled bluntly, invite overgeneralization and reinforce stereotypes (D'Orazzi and Hajek, 2026). The integrative move taken here is intended to guard against this: because cultural content is compared, enacted, and reflected on rather than delivered as fact, the emphasis falls on interpretation and self-relativization rather than on cataloging difference. Comparison in the language classroom is productive only when it foregrounds intra-cultural variation and treats the learner’s own assumptions as equally open to examination; otherwise, it can entrench the very stereotypes intercultural competence is meant to dissolve and leave ethnocentric positions intact. Because moving beyond an ethnocentric standpoint is itself a gradual, biographically situated process rather than an automatic result of exposure (D'Orazzi, 2026b), tasks here are designed so that learners diagnose and repair their own interpretive defaults rather than memorize cultural rules—which is what keeps cultural comparison from hardening into cultural stereotype.

The evidence base for this approach in Chinese-as-a-second-language settings, though, remains more oriented toward description and teacher perspective than toward learner effects (Dai et al., 2025; Gong et al., 2022; Li, 2025). Gong et al. (2022) documented how preservice Chinese language teachers conceive of intercultural teaching, and Dai et al. (2025) profiled Mandarin learners’ competence in one national setting, yet neither was built to test whether a specified program changes competence. The practical value of these studies is real; the point is that they leave the learner-level, mechanism-level question open.

### 2.3 Intercultural willingness to communicate as a psychological mechanism

Willingness to communicate captures a learner’s readiness to enter communication when free to do so—a state shaped by confidence and affiliation, not a count of classroom turns (MacIntyre et al., 1998). In intercultural settings, it names the disposition to speak, ask, explain, and negotiate across cultural differences, the very terrain on which competence is exercised. Recent work has treated this readiness as sensitive to affect, motivation, self-concept, and the texture of classroom interaction: ideal-self and grit, enjoyment and its absence, and the perceived quality of social interaction have each been tied to willingness to communicate (Ebn-Abbasi et al., 2024; Ebn-Abbasi and Nushi, 2022; Han and Li, 2025; Huang and Zhang, 2025; Lan et al., 2021; Li, 2024; Yang et al., 2024). Han and Li's (2025) finding that willingness varies by interactional activity type is directly relevant here, since it suggests that task design can move the variable.

If willingness responds to interactional conditions, culturally integrated tasks are a plausible lever. Cultural comparison, role-play, and short reflections reduce the strangeness of intercultural exchange, give learners rehearsed footholds, and provide a comparatively safe setting to try meaning-making—conditions that should increase readiness to communicate. Willingness, in turn, has been associated with intercultural competence and communicative outcomes: learners more prepared to engage are more likely to participate, negotiate, and build the reflective understanding that competence requires (Buelt, 2025; Choi, 2024; Fathi et al., 2023; Miauw and Guo, 2021; Wang and Cionea, 2024). Buelt's (2025) analysis linking motivation and willingness to communicate to intercultural competence development illustrates the pathway this study formalizes, though it does so in a different educational system.

Evidence specific to Chinese-as-a-second-language learners is emerging but young. Lv et al. (2021) connected pragmatic competence and willingness to communicate among L2 Chinese learners, and more recent study has begun to examine willingness to communicate more directly (Li et al., 2025; Li, 2025). The trajectory is clear enough to justify positioning intercultural willingness to communicate as a candidate mechanism, while the thinness of the evidence is itself part of the case for testing it.

### 2.4 Research hypotheses and conceptual model

The reasoning above yields a compact model in which instruction acts on competence both directly and through willingness to communicate. Prior intervention work on culturally integrated and experiential designs supports the expected direction of the instructional effect on competence (Huang, 2021; Lee et al., 2023; Liu, 2022; Wibowo et al., 2024). Interactive, technology-supported, and socially rich tasks support the expected effect on willingness to communicate (Choi, 2024; Fathi et al., 2025; Han and Li, 2025; Huang and Zhang, 2025). The competence–willingness association motivates the predictive path from willingness to competence (Fathi et al., 2023; Miauw and Guo, 2021; Wang and Cionea, 2024). The broader practice of using a psychological state to explain instructional or communicative outcomes in second language research supports modeling willingness as a mediator—an analogical warrant, since these studies address related but not identical constructs (Brauer et al., 2023; Fathi et al., 2023; Fu, 2025; Huang, 2026; Zhu, 2026).

*H1*: Learners receiving culturally integrated Mandarin instruction will show greater improvement in overall intercultural communicative competence than learners receiving regular Mandarin instruction.

*H2*: Learners receiving culturally integrated Mandarin instruction will show greater improvement in overall intercultural willingness to communicate than learners receiving regular Mandarin instruction.

*H3*: Intercultural willingness to communicate will positively predict post-intervention intercultural communicative competence after controlling for baseline competence.

*H4*: Intercultural willingness to communicate will statistically account for part of the association between culturally integrated Mandarin instruction and intercultural communicative competence.

The conceptual model links instruction type to intercultural willingness to communicate, willingness to intercultural communicative competence, and instruction directly to competence; the estimated model, with path coefficients, appears with the mediation results. Given the quasi-experimental, single-occasion design, these hypotheses are examined as associations rather than as tests of strict causal effects.

## 3 Methods

### 3.1 Research design

The study used a quasi-experimental pre-test/post-test control-group design, a common choice for evaluating instructional interventions in language education where random assignment of intact classes is impractical (Lee et al., 2023; Wibowo et al., 2024; Xu, 2023). The experimental group received culturally integrated Mandarin instruction and the control group received regular Mandarin instruction over the same 8-week period, with a weekly 90-min session in each condition. The study collected course feedback, weekly reflections, and short interviews to help interpret the quantitative pattern; they served as supplementary explanations, and the study is not presented as a full mixed-methods design. The same instructor taught both conditions, they met for the same weekly duration, and they used the same core Mandarin materials, with cultural integration as the systematic difference between them. The assignment operated at the level of one intact class per condition, so condition and class coincide, and class-level factors cannot be separated statistically from the instructional contrast. Outcomes were self-reported, and the mediator and outcome were measured at the same post-test occasion, so the design does not establish temporal precedence between willingness and competence. The design therefore supports claims about association and plausible mechanism rather than strict causal proof.

### 3.2 Participants and context

Recruitment yielded 82 learners studying Mandarin as a second language in intact classes at the same institution. They varied in nationality and first-language background, ranged in age from the late teens to the mid-twenties, and reported approximately 1 to 2 years of prior Mandarin study, with placement spanning HSK3 to intermediate levels. Both women and men participated, and a small number preferred not to report their gender. Prior intercultural experience ranged from none to extensive.

The study was approved by the institutional ethics committee of Zhejiang Normal University (approval number: IRB-2025-275), and all participants provided written informed consent before data collection, in accordance with the Declaration of Helsinki. Attrition and data matching across the two testing points, together with an attendance threshold of at least 75% of sessions, reduced the sample to the analytic set used for all reported models. Figure 1 shows the recruitment-to-analysis funnel and the design timeline.

**Figure 1:**
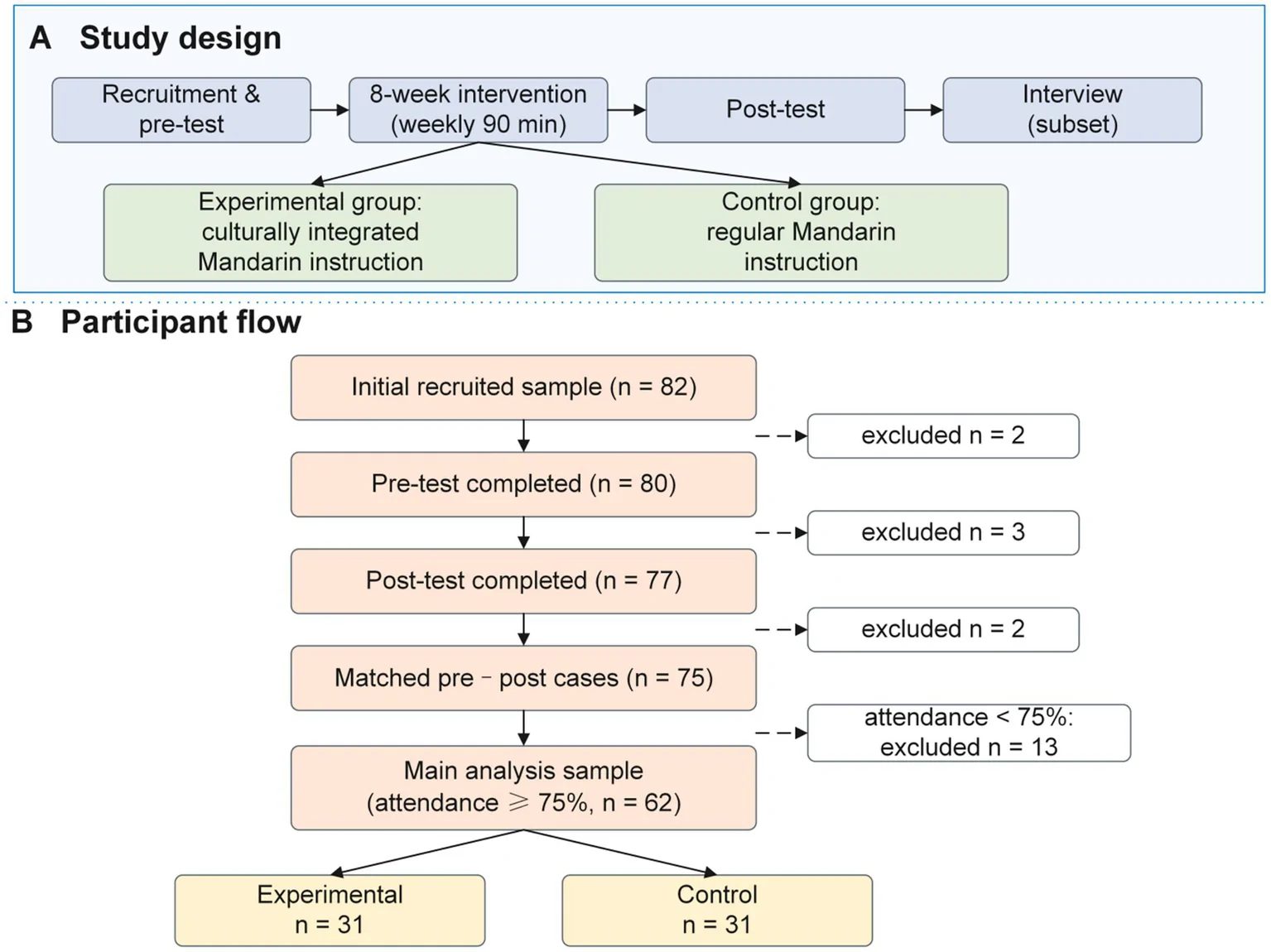
Research design and participant flow. **(B)** Initial recruited sample = 82 → pre-test completed = 80 → post-test completed = 77 → matched pre–post cases = 75 → main analysis sample with attendance ≥ 75% = 62 (experimental *n* = 31; control *n* = 31). **(A)** The flow is arranged left to right across the design phases: recruitment and pre-test, 8-week intervention, post-test, and post-test interviews with a purposively selected subset.

Losing 20 of the original 82 learners before analysis is a non-trivial reduction, and the attendance criterion was applied deliberately so the estimated effects reflect learners who received a meaningful dose of instruction rather than nominal enrollees. The equal final cell sizes—31 per group—also simplify the group comparisons that follow and reduce the risk that an imbalance in group size distorts the adjusted estimates.

### 3.3 Intervention design

The experimental program organized cultural content into communicative tasks across 8 weekly units, moving from culture in Mandarin communication through address and politeness, food and hospitality, festivals, indirect meaning, personal narrative, and intercultural problem-solving, to a final cultural comparison presentation. Each unit paired a Mandarin language focus with an intercultural task—cultural comparison, small-group discussion, role-play, misunderstanding-repair, or short reflection—so learners worked with cultural material rather than being delivered it. Two units illustrate the design. In the unit on address forms and politeness, the language focus was address terms and polite requests; learners role-played the same request to a teacher, a peer, and a stranger, compared how relationship and status reshaped the wording, and then reflected in writing on how their first-language norms had guided their initial choices. In the unit on indirect meaning, the language focus was suggestion and refusal expressions; learners worked through a scenario in which a direct refusal caused offense, enacted alternative face-saving formulations, and traced where the misunderstanding had originated. In each unit, task revealed the cultural point rather than stating it in advance. The control group covered comparable Mandarin content through vocabulary, grammar, and reading and speaking exercises, without systematic intercultural tasks. The two conditions were as similar as possible in instructional time, teaching staff, and core materials, so cultural integration, rather than teaching intensity, was the systematic difference. The full unit-by-unit outline—giving, for each week, the language focus, the cultural focus, the experimental task, and the targeted competence and willingness dimensions—appears in Supplementary Table 1. Two principles governed the sequence. Task complexity rose only as learners’ Mandarin allowed, so that intercultural demands stayed within their linguistic resources. Each week moved from noticing, through enactment, to reflection, so comparison ended by relating cultural difference to the learners’ own assumptions rather than listing features. By holding language focus, cultural focus, and task type constant while adjusting the difficulty of the target expressions to a class’s proficiency, the sequence can be reused in other Mandarin classrooms.

### 3.4 Measures

The two focal constructs were measured with scales developed for this study; each worked established theoretical frameworks and the dimensional logic of related tools into the specific setting of Mandarin learners’ classroom communication. Both used a 5-point Likert format (1 = strongly disagree to 5 = strongly agree), with total and dimension scores computed as item means and higher scores indicating a higher standing on the dimension. The intercultural communicative competence scale contained 20 items across four dimensions of five items each and, consistent with the study’s focus on intercultural verbal communication, its items concern language-based intercultural interaction rather than non-verbal channels. It drew on Byram's (1997) model of intercultural communicative competence, Deardorff's (2006) competence model, and the measurement thinking behind the Intercultural Sensitivity Scale (Chen and Starosta, 2000), without adopting that scale directly or paring its 24 items down to 20; items were reorganized around cultural understanding, comparison, and adjustment in Mandarin classroom situations. The four dimensions cover cultural awareness (noticing cultural difference, context, and the implicit cultural meaning carried in Mandarin interaction), intercultural attitude (openness, respect, and an effort to understand across cultural difference), interpretation and comparison skills (explaining Mandarin communicative phenomena and relating them to one’s own cultural experience), and interaction and adjustment skills (adjusting expression, responding to difference, and repairing misunderstanding in real or simulated exchange). The intercultural willingness to communicate scale contained 12 items across three dimensions of four items each, built on the situational model of L2 willingness to communicate (MacIntyre et al., 1998), which links readiness to communicate to learners’ wider motivation for language study (D'Orazzi, 2026a), and framed for Mandarin classroom tasks, intercultural interaction, and uncertain communicative situations rather than adapted from any fixed validated willingness-to-communicate instrument. Its dimensions cover willingness to initiate interaction (starting intercultural exchange in Mandarin on one’s own), willingness to participate in classroom intercultural tasks (joining cultural discussion, role-play, group exchange, and intercultural activities), and willingness to manage uncertainty (still attempting expression and sustained interaction when vocabulary is thin, cultural understanding is partial, or the outcome is unsure). Item wording throughout kept the willingness scale on readiness to communicate rather than on communicative ability itself.

Development followed content review and pilot testing. Two experts reviewed the questionnaire—one with a background in Chinese language education, the other in intercultural communication research—and their comments concentrated on two points: making the communicative situation in each item concrete, and separating willingness to communicate from the ability to communicate so that competence judgments would not leak into the willingness items. Wording was revised accordingly, adding classroom, cultural-discussion, peer-exchange, and intercultural-task contexts and keeping the willingness items on intention and initiative. A pilot with 26 respondents checked item clarity, scale length, completion time, and whether learners could follow the situations described; a few culture-referenced items were then made more specific. Items were presented in English, with Chinese equivalents retained in the research record and in online administration on a five-point scale, so that a learner encountering an unfamiliar English item could consult the Mandarin gloss. A formal translation and back-translation procedure was not carried out; therefore, the scales are not a validated bilingual version, and variation in item comprehension with English proficiency remains possible. Given the limited sample, internal consistency is the primary psychometric evidence, supported by the expert review and pilot. Internal consistency was indexed by both Cronbach’s alpha and McDonald’s omega, which agreed closely at the total-scale level, and corrected item–total correlations were positive across items (Supplementary Table 3). Within-construct dimension correlations were on the whole higher than between-construct correlations (Supplementary Table 4), though the margin was small in places—the highest between-construct correlation (approximately 0.72) fell only marginally below the lowest within-construct correlation (approximately 0.73). No exploratory or confirmatory factor analysis was conducted; the scales carry evidence of content, reliability, and a preliminary pattern of discriminant correlations, and not of confirmed factorial structure. Total scores served as the outcome variables throughout, because the total scales showed acceptable-to-strong internal consistency, while several willingness-to-communicate subdimensions were weaker at pre-test, which would make dimension-level effects less stable. All inferential analyses used total scores; dimension-level pre–post values were treated as descriptive only and are reported, without inferential testing, in Supplementary Table 5. Pre- and post-test totals fed the group comparisons, change analyses, analysis of covariance, and the mediation model. Researchers recorded background variables—age, gender, length of Mandarin learning, Mandarin proficiency, and prior intercultural experience—for baseline comparison, and short semi-structured interviews probed learners’ sense of change in cultural understanding, willingness to communicate, and task experience.

### 3.5 Data collection procedure

Pre-test questionnaires were administered before the intervention began, and identical post-test questionnaires were administered after the 8-week program ended. Semi-structured interviews were conducted after the post-test with eight learners, purposively selected to span the range of ICC and IWTC change profiles (from larger to smaller pre-to-post gains) so that varied experiences of the program would be represented. Interview duration was not systematically recorded. Questionnaire responses were matched across the two time points by anonymized identifiers and then de-identified for analysis, with interview records anonymized before coding. Testing conditions were kept consistent across groups to limit administration effects.

### 3.6 Data analysis

Analysis proceeded from description to inference. Descriptive statistics summarized each group at each time point; internal consistency was estimated with both Cronbach’s alpha and McDonald’s omega, complemented by corrected item—total correlations and an inspection of within- versus between-construct dimension correlations; baseline equivalence was tested across demographic and outcome variables. Within-group pre–post change was described with paired-samples *t*-tests, and between-group effects were evaluated with change-score comparisons and analysis of covariance adjusting for the corresponding baseline score as well as length of Mandarin learning and Mandarin proficiency. The mediating role of intercultural willingness to communicate was tested with regression-based path analysis and bootstrap confidence intervals for the indirect effect, following established guidance for indirect-effect estimation and quantitative practice in applied linguistics (Hair and Alamer, 2022; Preacher and Hayes, 2008); a full structural equation approach was deliberately avoided given the sample size. Common method variance was examined with Harman’s single-factor test on the pre-test items for the 47 learners with complete responses to all items (listwise deletion); this test is a limited diagnostic and cannot rule out common method bias. The indirect effect was re-estimated under full-covariate, no-covariate, and baseline-only specifications, each with percentile bootstrap intervals from 5,000 resamples, to assess its stability across covariate sets. Interview and reflection data were analyzed thematically by a single coder: transcripts and reflective texts were read for recurring content, assigned initial codes, and grouped into higher-order themes, with theme and initial-code frequencies retained. The coding was carried out by one researcher, without a second coder, so inter-coder reliability was not established; the interview and reflection material serves an illustrative and interpretive purpose, adding texture to the quantitative pattern rather than providing independent triangulation.

The mediation models were estimated as ordinary least squares path regressions in Python, with the indirect effect assessed through non-parametric bootstrapping (5,000 resamples; random seed 10,097), which reproduces the logic of a single-mediator model without relying on latent-variable software. The mediator model regressed post-test intercultural willingness to communicate on group membership, controlling for pre-test willingness to communicate, pre-test competence, length of Mandarin learning, and Mandarin proficiency. The outcome model regressed post-test competence on group membership and post-test willingness to communicate, controlling for pre-test competence, pre-test willingness to communicate, length of Mandarin learning, and Mandarin proficiency. The total-effect model used the same covariates as the outcome model but omitted the mediator. Cases with missing values on any variable entering a given model were removed listwise; neither full-information maximum likelihood nor imputation was applied.

Because the analysis of covariance and the mediation models do not share an identical covariate set, the group effect on competence appears as three slightly different values. The unadjusted post-test difference is approximately 0.520; the analysis of covariance, controlling for the matching baseline score, learning duration, and proficiency, yields approximately 0.530; and the total-effect model, which also controls for pre-test willingness to communicate, yields approximately 0.603. The upward shift reflects covariate adjustment rather than instability: the experimental group began slightly lower on willingness to communicate, so holding that baseline constant raises the estimated group effect on competence. The group effect on willingness likewise appears as two values—approximately 0.590 in the analysis of covariance and approximately 0.635 in the mediator model—because the mediator model additionally controls for pre-test competence; the difference again reflects covariate adjustment rather than instability.

Internal consistency was computed as (Equation 1):


$$\alpha =\frac{k}{k-1}(1-\frac{\sum_{i=1}^{k}\sigma_{i}^{2}}{\sigma_{T}^{2}})$$ (1)

Change was defined as (Equation 2):


$$\mathrm{\Delta Y}=Y_{\text{post}}-Y_{\mathrm{pre}}$$ (2)

The adjusted group comparison took the form (Equation 3):


$$Y_{\text{post}}=\beta_{0}+\beta_{1}\text{Group}+\beta_{2}Y_{\mathrm{pre}}+\epsilon$$ (3)

and the mediated (indirect) effect was estimated as (Equation 4):


ab=a×b (4)

where a is the instruction-to-willingness path and b the willingness-to-competence path.

## 4 Results

### 4.1 Participant flow, reliability, and baseline equivalence

Baseline characteristics and equivalence tests across the two conditions are reported in Table 1.

**Table 1:** Participant characteristics and baseline equivalence.

Variable	Experimental	Control	Test
Age	21.71 ± 1.90	21.16 ± 1.85	*t* = 1.15, *p* = 0.254
Learning duration	17.65 ± 5.45	18.09 ± 4.59	*t* = −0.34, *p* = 0.735
Pre-test ICC	2.96 ± 0.50	3.16 ± 0.41	*t* = −1.71, *p* = 0.093
Pre-test IWTC	2.86 ± 0.43	2.97 ± 0.45	*t* = −1.03, *p* = 0.307
Gender	Female 18, Male 13	Female 19, Male 12	Fisher exact, *p* = 1.000
Mandarin level	HSK3 9; HSK4 9; HSK5 5; Intermediate I 6; Intermediate II 2	HSK3 3; HSK4 14; HSK5 5; Intermediate I 3; Intermediate II 6	χ^2^ = 7.09, *p* = 0.131
Prior intercultural experience	High 7; Low 7; Moderate 10; Missing 7	High 4; Low 11; Moderate 9; Missing 7	χ^2^ = 1.76, *p* = 0.415

Characteristics are reported for the analytic sample (*N* = 62; experimental *n* = 31, control *n* = 31). Prior intercultural experience was self-reported and optional; missing responses (7 per group) were retained as a separate category and this variable was used only for baseline description, not entered into any inferential model.

The groups were statistically indistinguishable at baseline on age, learning duration, gender, Mandarin level, prior intercultural experience, and both outcome measures. The one value that comes close to a difference is pre-test ICC, where the control group sat slightly higher (*t* = −1.71, *p* = 0.093). That near-miss is why the primary group comparisons rely on covariate adjustment rather than raw post-test contrasts: if left uncontrolled, the control group’s small head start would bias against the experimental group and understate any true effect.

Because 20 of the 82 recruited learners were not carried into the analytic sample, retained and non-retained learners were compared on baseline characteristics (Table 2). Of these 20, two did not complete the pre-test, and the remaining 18 completed it but were not included in the final analysis; the baseline comparison therefore draws on the 80 pre-test completers, of whom 62 entered the analytic sample, and 18 did not. Retention was close across conditions, at 79.5% in the control group (31 of 39) and 75.6% in the experimental group (31 of 41), χ^2^(1) = 0.02, *p* = 0.883. Retained and non-retained learners did not differ in age, length of Mandarin learning, Mandarin level, prior intercultural experience, or pre-test competence. Two baseline characteristics did differ: pre-test willingness to communicate was higher among retained learners (M = 2.92, SD = 0.44) than among those not retained (M = 2.67, SD = 0.37), *t* = 2.39, *p* = 0.023, and the distribution of gender differed by retention status, χ^2^(2) = 7.52, *p* = 0.023. Attrition was balanced across the two instructional conditions, while the retained sample had slightly higher baseline willingness than the initial pool.

**Table 2:** Baseline comparison of retained and non-retained learners.

Measure	Retained (*n* = 62)	Not retained (*n* = 18)	Test
Age	21.44 ± 1.88	21.44 ± 2.38	*t* = −0.01, *p* = 0.988
Length of Mandarin learning	17.87 ± 5.00	17.93 ± 6.29	*t* = −0.04, *p* = 0.970
Pre-test ICC	3.06 ± 0.46	3.24 ± 0.38	*t* = −1.69, *p* = 0.100
Pre-test IWTC	2.92 ± 0.44	2.67 ± 0.37	*t* = 2.39, *p* = 0.023
Gender	Female 37, male 25	Female 8, male 8, prefer not to say 2	χ^2^(2) = 7.52, *p* = 0.023
Mandarin level	HSK3 12; HSK4 23; HSK5 10; intermediate I 9; intermediate II 8	HSK3 5; HSK4 5; HSK5 3; Intermediate I 4; Intermediate II 1	χ^2^(4) = 1.99, *p* = 0.737
Prior intercultural experience	High 11; low 18; moderate 19; missing 14	High 1; low 6; moderate 6; missing 5	χ^2^(3) = 1.66, *p* = 0.646

Baseline comparisons and retention rates were computed on the 80 learners who completed the pre-test. Retention was 79.5% in the control group (31 of 39) and 75.6% in the experimental group (31 of 41), χ^2^(1) = 0.02, *p* = 0.883.

Reliability coefficients for both measures are reported in Table 3.

**Table 3:** Reliability coefficients of ICC and IWTC measures.

Scale	Items	Pre-test α	Post-test α
ICC total	20	0.885	0.939
Cultural awareness	5	0.713	0.788
Intercultural attitude	5	0.614	0.854
Interpretation and comparison skills	5	0.701	0.779
Interaction and adjustment skills	5	0.665	0.842
IWTC total	12	0.776	0.880
Willingness to initiate interaction	4	0.609	0.697
Willingness to participate in classroom intercultural tasks	4	0.543	0.767
Willingness to manage uncertainty	4	0.434	0.751

Both total scales reached acceptable or strong internal consistency at both time points, rising from pre-test to post-test. The subscale coefficients are weaker, and two willingness-to-communicate subdimensions fall below the usual threshold at pre-test—participation in classroom intercultural tasks (0.543) and managing uncertainty (0.434). All inferential analyses therefore used total scores, keeping estimates away from the least stable components; the subscale coefficients are reported in full for transparency. Scale scores used throughout were computed as the mean of answered items for the full analytic sample, whereas reliability coefficients were estimated on learners with complete item-level data for each scale.

### 4.2 Group differences in intercultural communicative competence

Testing H1 requires both the within-group change for each condition and a between-group comparison that accounts for the baseline gap noted above. Table 4 reports pre-test, post-test, and change scores for both outcomes, and Table 5 reports the covariate-adjusted group effects.

**Table 4:** Pre-test, post-test, and change scores for ICC and IWTC.

Outcome	Group	*n*	Pre M ± SD	Post M ± SD	Change M ± SD	Paired *t*	*p*	d
ICC	Experimental	31	2.958 ± 0.496	3.650 ± 0.653	0.692 ± 0.835	4.62	< 0.001	0.83
ICC	Control	31	3.155 ± 0.410	3.130 ± 0.661	−0.026 ± 0.787	−0.18	0.858	−0.03
IWTC	Experimental	31	2.859 ± 0.434	3.519 ± 0.473	0.660 ± 0.549	6.70	< 0.001	1.20
IWTC	Control	31	2.974 ± 0.451	2.961 ± 0.544	−0.014 ± 0.623	−0.12	0.903	−0.02

Change-score comparison between groups					
Change score	Experimental	Control	*t*	*p*	d
ICC change	0.692 ± 0.835	−0.026 ± 0.787	3.48	0.001	0.88
IWTC change	0.660 ± 0.549	−0.014 ± 0.623	4.52	< 0.001	1.15

**Table 5:** ANCOVA results for post-test ICC and IWTC.

Outcome	Group estimate	SE	*p*	R^2^	*n*
Post-test ICC	0.530	0.179	0.005	0.179	62
Post-test IWTC	0.590	0.129	< 0.001	0.330	62

For intercultural communicative competence, the experimental group increased from pre to post, while the control group essentially held still, and the between-group change comparison favored the experimental condition with a large effect size. The covariate-adjusted estimate points the same way: after controlling for baseline ICC, group membership predicted higher post-test competence (0.530, *p* = 0.005). The three views of the same data—within-group gain, between-group change, and adjusted comparison—point in the same direction, so the ICC advantage is unlikely to be an artifact of the small baseline imbalance. The model’s modest R^2^ for post-test ICC also indicates that competence is associated with more than group membership alone, consistent with a mediated account.

### 4.3 Group differences in intercultural willingness to communicate

The same reporting logic applies to willingness to communicate, testing H2. The relevant values appear in the willingness rows of Table 4 and the willingness row of Table 5. The experimental group rose from a pre-test mean of 2.859 to a post-test mean of 3.519, a within-group change with a very large effect size, while the control group did not move. The between-group change comparison strongly favored the experimental condition, and the covariate-adjusted estimate was consistent with a higher post-test willingness score for that group (0.590, *p* < 0.001). The adjusted model accounted for more variance in willingness than in competence, consistent with the mediation analysis that follows: instruction was more closely associated with the proposed mechanism than with the distal outcome.

### 4.4 Mediation analysis

The remaining questions concern whether willingness predicts competence net of baseline (H3) and whether it accounts for the association between instruction and competence (H4). The path estimates and the bootstrap indirect effect are reported in Table 6 (Figure 2).

**Table 6:** Mediation analysis results.

Path/effect	Estimate	SE	*p*	95% CI
Group → post-IWTC	0.635	0.135	< 0.001	—
Post-IWTC → post-ICC	0.819	0.129	< 0.001	—
Direct effect, group → post-ICC	0.083	0.153	0.590	—
Total effect, group → post-ICC	0.603	0.169	0.001	—
Indirect effect	0.520	0.148	—	[0.256, 0.823]

Model summaries			
Model	R^2^	Adj. R^2^	*p*
Mediator model	0.346	0.287	< 0.001
Outcome model	0.596	0.552	< 0.001
Total effect model	0.298	0.235	0.001

**Figure 2:**
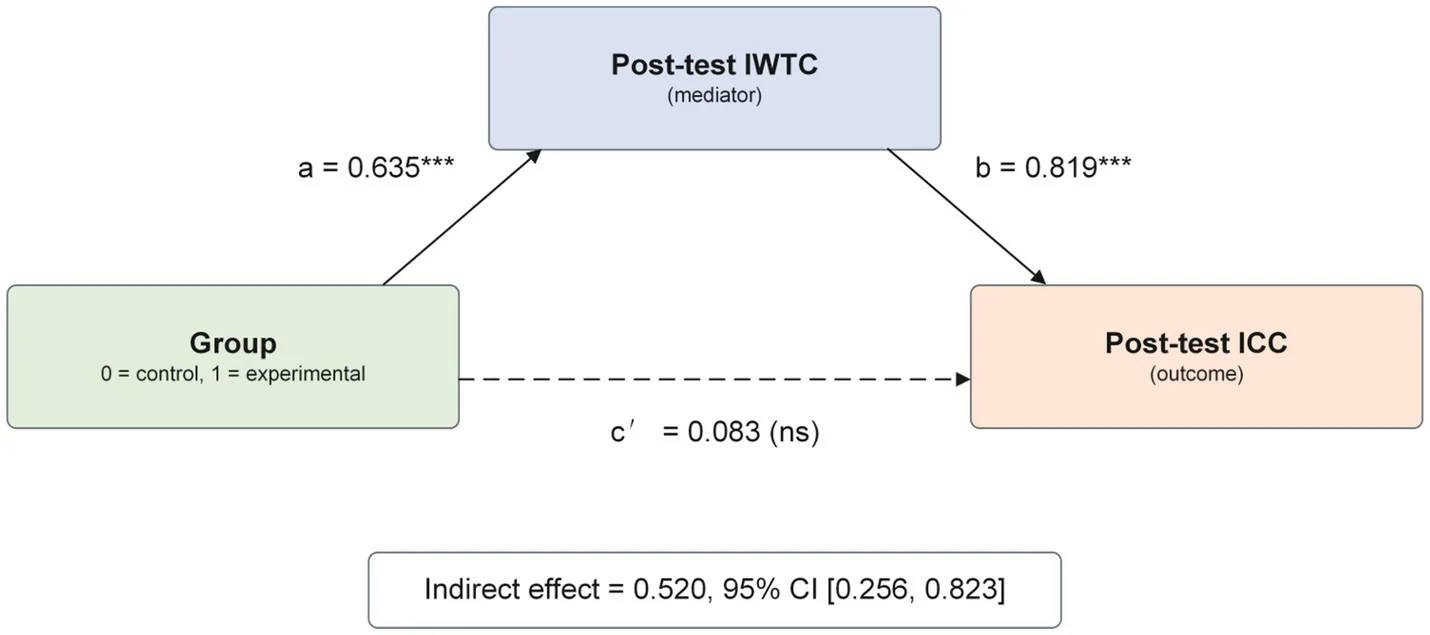
Mediation model with path coefficients. Group → post-IWTC = 0.635; post-IWTC → post-ICC = 0.819; direct group → post-ICC = 0.083 (ns); indirect effect = 0.520, 95% CI [0.256, 0.823]. ***p < 0.001.

The path from instruction to willingness was positive and strong, as was the path from willingness to competence, while the direct path from instruction to competence was small and non-significant once willingness entered the model. The total effect of instruction on competence (0.603) was significant; when decomposed, almost all of it was associated with willingness (indirect effect 0.520), with a 95% confidence interval that excluded zero, leaving only a negligible direct path. The higher explained variance of the outcome model relative to the total-effect model reflects the added predictive contribution of willingness. Read together, the coefficients are consistent with a mediating role for willingness to communicate. Because willingness and competence were measured at the same occasion, this pattern is equally compatible with the reverse ordering, in which rising competence supports willingness, and the size of the indirect effect is best read as an upper bound rather than a literal decomposition.

A Harman single-factor test was run on the pre-test items (*n* = 47 with complete item-level responses) to gauge common method variance. Eleven factors had eigenvalues above one, and the first unrotated factor accounted for 20.1% of the variance, so a single method factor does not dominate the item set, though this test is only a limited diagnostic. The indirect effect was stable across covariate specifications. It was 0.520, 95% CI [0.256, 0.823], with the full covariate set; 0.494, 95% CI [0.255, 0.765], with no covariates; and 0.535, 95% CI [0.292, 0.829], with baseline scores only. All three percentile bootstrap intervals excluded zero.

### 4.5 Course feedback and qualitative supplement

Feedback and reflection data are summarized in Supplementary Table 2. Course feedback item means ranged from 3.87 to 4.18, and weekly reflection submissions stayed high across the eight units (28, 28, 33, 33, 35, 33, 37, 34; these counts reflect all enrolled experimental-group learners submitting in a given week, not the 31-learner analytic sample). In the reflections, references to “culture” (316) were most frequent, followed by “different” (124), “polite” (86), “question” (62), “willing” (53), and “role play” (53). Interview coding of the eight interviews by a single coder surfaced four recurring themes: cultural awareness (14), task-based confidence (10), remaining language constraint (9), and increased willingness (7), built up from initial codes that included role-play support (9), vocabulary difficulty (9), confidence growth (8), cultural comparison (7), and uncertainty management (7). The prominence of willingness- and confidence-related themes alongside the frequency of “culture” and “question” consistent with the pattern in the outcome data, and the qualitative material is read here as illustrative rather than as independent confirmation.

## 5 Discussion

The study aimed to determine whether culturally integrated Mandarin instruction improves learners’ intercultural communicative competence and willingness to communicate, and whether willingness explains any competence gain. Across three convergent tests, the experimental group improved on both outcomes while the control group did not, and the adjusted comparisons held after accounting for a slight baseline difference. The bootstrap mediation was consistent with willingness to communicate as the route from instruction to competence, with the direct path fading to non-significance once willingness was included. This overall shape echoes recent research linking intercultural competence to communicative readiness, though the present design adds a learner-level test in a Mandarin context (Fathi et al., 2023; Lee et al., 2023).

The competence gain is best read not as an increase in cultural information but as growth in the integrated capacity to interpret, engage, and adjust. Placing cultural comparison, small-group discussion, role-play, misunderstanding-repair tasks, and short reflections at the center of language tasks gave learners repeated occasions to make and repair culturally loaded meaning, not merely to receive accounts of it, the kind of experience that competence research associates with development (Huang, 2021; Lee et al., 2023; Liao, 2024; Liu, 2022; Reader, 2024; Wibowo et al., 2024). This reading fits the data better than treating culture as a detachable content block layered onto ordinary instruction: the control group covered comparable Mandarin content without the intercultural tasks and did not improve. This is plausible but bounded by the design: the data support that the integrated task structure, not cultural exposure itself, was associated with the change. At the descriptive level, the experimental group showed gains across competence dimensions, including intercultural attitude, where it rose while the control group did not (Supplementary Table 5); these dimension-level values are descriptive only, given the weaker subscale reliability.

Willingness to communicate may plausibly account for this pattern, given what the tasks did to the interactional situation. Cultural comparison, role-play, and short reflections lowered the strangeness of intercultural exchange and offered a comparatively safe, structured setting in which to try meaning-making—conditions that plausibly reduced uncertainty and raised learners’ readiness to ask, explain, and negotiate in Mandarin (Han and Li, 2025; MacIntyre et al., 1998). The learners’ own records point to the same few activities: role-play and cultural comparison recurred among the initial codes drawn from the interviews (role-play support and cultural comparison), alongside confidence growth, while references to culture and to role-play were among the most frequent in the weekly reflections. The convergence is tentative, resting on frequency counts from a single coder, but it locates the active ingredients in the enacted and comparative tasks rather than in cultural exposure alone. Once more willing to engage, learners were positioned to participate in the interaction and reflection through which competence develops (Fathi et al., 2023; Li, 2025; Wang and Cionea, 2024; Yang et al., 2024). Willingness to communicate appears to carry most of the instructional effect; this is a plausible path, not an established mechanism. Willingness and competence were measured at the same post-test point, so the model cannot show that a rise in willingness preceded the rise in competence; the reverse ordering, in which improving competence made learners more willing to communicate, fits the same correlational structure just as well, and the present data cannot separate the two. The two constructs also overlap in content, and learners reported both in a single sitting. Under those conditions, shared method variance can inflate the willingness-to-competence path, so its size should not be read too literally. Beyond this, the near-total transmission through the indirect path leaves other mechanisms untested, including self-efficacy, motivation, and anxiety, that were not modeled, and a non-significant direct effect in a modest sample is weaker evidence than a direct comparison of competing mediators would provide.

The gains were large for an 8-week, once-weekly program, with effect sizes approximately 0.8 to 1.2. Several design features could inflate them: outcomes were self-reported, the experimental course was salient and novel, and learners rated themselves at a single post-test point. Two observations temper a purely artifactual reading—the control group, tested identically, did not move on either measure, and the free-response record tracked the measured pattern—but they do not remove it. The effect sizes are therefore best treated as upper-bound estimates, to be checked against objective or delayed indicators before they are read as the typical magnitude of such a program.

Positioned against the existing literature, the contribution is to move Chinese-as-a-second-language intercultural research from teacher cognition and descriptive accounts toward a learner-level intervention with an explicit mechanism (Dai et al., 2025; Gong et al., 2022; Li et al., 2025; Li, 2025; Lv et al., 2021). While earlier studies established how practitioners think about intercultural teaching and how learner attributes covary, these results link a defined program to competence gains and willingness to communicate as the pathway. The results speak to how learning, communication readiness, and competence develop, not to language promotion.

For teaching, the practical implication is to design cultural content as communicative tasks rather than background knowledge, and to treat willingness to communicate as a target in its own right, not only as something learners can or cannot do. Five formats used here place learners in real communicative positions and can be reused directly. Cultural comparison asks learners to set a Mandarin communicative norm beside one from their own background and account for the difference, rather than to memorize it. Small-group discussion turns a cultural point into a task learners must talk through to complete it. Role-play has learners enact the same exchange under shifting relationships or expectations, so they make politeness and framing choices. Misunderstanding-repair tasks give learners a scenario in which an intercultural exchange has gone wrong and ask them to diagnose and re-do it. Short reflections close each unit by asking learners to connect what they noticed to their own assumptions (Choi, 2024; Fathi et al., 2025; Liu, 2022; Peraza and Furumura, 2022; Yang and Samat, 2023). Task difficulty needs to match learners’ Mandarin level, since cultural materials that are too long or too demanding can undercut the very engagement they are meant to promote, a caution supported by the recurrence of vocabulary difficulty among the interview codes; the aim is participation, not coverage.

Several limitations bound these conclusions. The sample was modest and drawn from intact course groups, with one class per condition, so class and teacher effects coincide with the instructional contrast and cannot be separated statistically, and the quasi-experimental design supports association and plausible mechanism rather than causal proof (Brauer et al., 2023). Retention was balanced across conditions, but retained learners had somewhat higher baseline willingness to communicate and differed in gender composition from those not retained, so the analytic sample does not fully represent the recruited pool. Outcomes rested on self-report, and the study could not be blinded: learners in the experimental condition knew they were taking a distinctive culture-focused course and then rated their own competence and willingness, so demand characteristics and expectancy may have contributed to the group difference. That threat is real. Several features, however, make it an unlikely full explanation. The control group, also aware of being studied, did not drift upward on either measure, which is hard to reconcile with a generic testing or attention effect. The qualitative record points the same way as the ratings without merely restating them—learners’ weekly reflections returned most often to culture, and then to noticing difference, politeness, questioning, and role-play, while the interview themes that recurred were sharpened cultural awareness and greater willingness to engage—so the free-response signal tracks the measured pattern rather than a uniform tendency to endorse the course. These signals were gathered inside the intervention and cannot settle the question on their own, which is why observer-rated or task-based indicators collected outside the self-report frame would strengthen future tests. Weaker subscale reliability led us to rely on total scores, which limits dimension-level inference (Hair and Alamer, 2022; Tiurikova, 2021). Because the scales were administered in English to Mandarin-as-a-second-language learners, without a formal translation and back-translation procedure, item comprehension may have varied with English proficiency; future work should validate a properly translated bilingual version. The 8-week window and lack of a delayed post-test leave durability untested, and because the mediator and outcome were measured together, establishing the temporal priority of willingness to communicate would require assessing it partway through the intervention rather than only at the end. The design also treated movement beyond an ethnocentric standpoint as a guiding aim rather than a measured outcome; it did not assess whether learners’ ethnocentric positioning actually shifted, and this remains for future work. The study also addressed only the verbal dimension of the construct: it did not measure intercultural non-verbal communication competence, and extending the design to non-verbal channels would provide a fuller picture of intercultural communicative competence (Yang, 2025). Future work could enlarge and diversify samples, add delayed measurement and behavioral or performance-based indicators of intercultural interaction, and test willingness alongside self-efficacy, motivation, and anxiety to see whether the mediated pattern holds when rival mechanisms are modeled together (Preacher and Hayes, 2008).

## 6 Conclusion

This study asked whether culturally integrated Mandarin instruction is associated with gains in learners’ intercultural communicative competence, and through what psychological route. Using a quasi-experimental design with matched pre- and post-testing, learners who received the culturally integrated program improved on both intercultural communicative competence and intercultural willingness to communicate, while learners in regular instruction did not, and the group advantage held after adjustment for baseline scores. The mediation analysis was consistent with an instructional effect on competence operating largely through willingness to communicate, with a significant indirect effect and a non-significant direct effect. Read cautiously, given a self-report, quasi-experimental design, the results fit the interpretation that building cultural content into communicative tasks may have contributed to competence by raising learners’ readiness to engage across cultural differences. For international Chinese language education, the practical message is to treat culture as material for interaction and to treat willingness to communicate as a teachable outcome in its own right.

## Data Availability

The original contributions presented in the study are included in the article/Supplementary material, further inquiries can be directed to the corresponding author.
