# Supplementary material for: Culturally integrated Mandarin instruction and intercultural communicative competence: the mediating role of intercultural willingness to communicate
Source: Front Psychol. 2026 Oct 26;17:1929538. doi: 10.3389/fpsyg.2026.1929538 (PMC13745654; doi:10.3389/fpsyg.2026.1929538)
Supplement: Supplementary file 1 [file Supplementary_file_1.DOCX]

**Supplementary Materials**

**Supplementary Table 1. Eight-week unit outline of the culturally integrated Mandarin program.**

| **Week** | **Lesson topic** | **Language focus** | **Cultural focus** | **Experimental activities** | **Control activities** | **Target ICC dimension** | **Target IWTC dimension** |
| --- | --- | --- | --- | --- | --- | --- | --- |
| 1 | Culture in Mandarin Communication | Opinion and comparison expressions | Cultural differences in communication | Case discussion; cultural self-introduction | Regular self-introduction and comparison practice | Awareness | Initiation |
| 2 | Address Forms and Politeness | Address terms and polite requests | Relationship and politeness norms | Role-play with teacher, peer, stranger | Address terms and request drills | Attitude | Task |
| 3 | Food and Hospitality | Ordering, inviting, accepting/refusing | Food and hospitality practices | Meal invitation scenario comparison | Ordering and invitation dialogue practice | Skills | Uncertainty |
| 4 | Festivals and Meanings | Time and custom description | Festivals and cultural meanings | Festival comparison poster | Festival vocabulary and text comprehension | Awareness | Task |
| 5 | Indirect Meaning | Suggestion and refusal expressions | Direct and indirect communication | Face and refusal role-play | Suggestion and refusal drills | Interaction | Uncertainty |
| 6 | Stories and Personal Narratives | Sequence markers and storytelling | Stories and cultural values | Personal cultural story sharing | Narrating personal experiences | Skills | Initiation |
| 7 | Intercultural Problem Solving | Explaining causes and clarifying | Misunderstanding repair | Misunderstanding solution design | Cause explanation and clarification practice | Interaction | Uncertainty |
| 8 | Final Cultural Comparison Presentation | Summary and presentation language | Integrated cultural comparison | Group presentation and Q&A | Regular topic presentation | Overall | Overall |

**Supplementary Table 2. Qualitative data summary: reflection keywords, interview themes, and initial codes.**

| **Category** | **Item** | **Count** |
| --- | --- | --- |
| Reflection keyword | willing | 53 |
| Reflection keyword | culture | 316 |
| Reflection keyword | polite | 86 |
| Reflection keyword | role play | 53 |
| Reflection keyword | vocabulary | 45 |
| Reflection keyword | different | 124 |
| Reflection keyword | question | 62 |
| Interview theme | Cultural awareness | 14 |
| Interview theme | Task-based confidence | 10 |
| Interview theme | Remaining language constraint | 9 |
| Interview theme | Increased willingness | 7 |
| Initial code | Role-play support | 9 |
| Initial code | Vocabulary difficulty | 9 |
| Initial code | Confidence growth | 8 |
| Initial code | Cultural comparison | 7 |
| Initial code | Uncertainty management | 7 |

*Note. Frequencies were produced by a single coder; the qualitative material serves an illustrative rather than a triangulating role. Interview themes and initial codes are based on eight interviewees.*

**Supplementary Table 3. Additional reliability evidence: McDonald's omega and corrected item–total correlations.**

| **Scale / Dimension** | **Items** | **n** | **McDonald's ω** | **Corrected item–total r (range)** |
| --- | --- | --- | --- | --- |
| Intercultural communicative competence total | 20 | 54 | 0.882 | 0.26 to 0.69 |
| — Cultural awareness | 5 | 60 | 0.743 | 0.40 to 0.66 |
| — Intercultural attitude | 5 | 60 | 0.633 | 0.29 to 0.47 |
| — Interpretation and comparison skills | 5 | 59 | 0.623 | 0.30 to 0.48 |
| — Interaction and adjustment skills | 5 | 59 | 0.617 | 0.20 to 0.54 |
| Intercultural willingness to communicate total | 12 | 54 | 0.794 | 0.33 to 0.60 |
| — Willingness to initiate interaction | 4 | 59 | 0.627 | 0.32 to 0.48 |
| — Willingness to participate in classroom tasks | 4 | 58 | 0.558 | 0.28 to 0.38 |
| — Willingness to manage uncertainty | 4 | 61 | 0.464 | 0.22 to 0.31 |

*Note. Coefficients were computed on respondents with complete item-level data for each (sub)scale; ns therefore vary. Scale scores used in all other analyses were computed as the mean of answered items for the full analytic sample (n = 62). McDonald's omega and corrected item–total correlations were computed on pre-test responses, reported as additional indices of internal consistency.*

**Supplementary Table 4. Inter-dimension correlation matrix (post-test).**

| **Dimension** | **1** | **2** | **3** | **4** | **5** | **6** | **7** |
| --- | --- | --- | --- | --- | --- | --- | --- |
| 1 Cultural awareness | 1.00 |  |  |  |  |  |  |
| 2 Intercultural attitude | 0.76 | 1.00 |  |  |  |  |  |
| 3 Interpretation and comparison | 0.73 | 0.73 | 1.00 |  |  |  |  |
| 4 Interaction and adjustment | 0.81 | 0.76 | 0.82 | 1.00 |  |  |  |
| 5 Willingness to initiate | 0.59 | 0.52 | 0.70 | 0.72 | 1.00 |  |  |
| 6 Willingness to participate in tasks | 0.53 | 0.58 | 0.59 | 0.66 | 0.76 | 1.00 |  |
| 7 Willingness to manage uncertainty | 0.53 | 0.49 | 0.63 | 0.69 | 0.73 | 0.73 | 1.00 |

*Note. Pearson correlations among post-test dimension mean scores, using pairwise complete observations.*

**Supplementary Table 5. Dimension-level pre/post descriptives by group (exploratory).**

| **Dimension** | **Group** | **n** | **Pre-test M ± SD** | **Post-test M ± SD** | **Change M ± SD** | **Paired d** |
| --- | --- | --- | --- | --- | --- | --- |
| Cultural awareness | Experimental | 31 | 2.90 ± 0.60 | 3.57 ± 0.70 | +0.67 ± 1.03 | 0.66 |
| Cultural awareness | Control | 31 | 3.12 ± 0.52 | 3.03 ± 0.57 | −0.09 ± 0.76 | −0.12 |
| Intercultural attitude | Experimental | 31 | 2.99 ± 0.60 | 3.69 ± 0.67 | +0.70 ± 0.88 | 0.79 |
| Intercultural attitude | Control | 31 | 3.26 ± 0.47 | 3.15 ± 0.79 | −0.11 ± 0.95 | −0.11 |
| Interpretation and comparison | Experimental | 31 | 3.01 ± 0.50 | 3.65 ± 0.73 | +0.64 ± 0.83 | 0.78 |
| Interpretation and comparison | Control | 31 | 3.08 ± 0.46 | 3.11 ± 0.78 | +0.03 ± 0.83 | 0.03 |
| Interaction and adjustment | Experimental | 31 | 2.94 ± 0.54 | 3.70 ± 0.77 | +0.75 ± 0.97 | 0.78 |
| Interaction and adjustment | Control | 31 | 3.15 ± 0.47 | 3.22 ± 0.83 | +0.07 ± 0.95 | 0.07 |
| Willingness to initiate | Experimental | 31 | 2.90 ± 0.52 | 3.49 ± 0.55 | +0.59 ± 0.69 | 0.85 |
| Willingness to initiate | Control | 31 | 3.01 ± 0.57 | 2.91 ± 0.61 | −0.09 ± 0.76 | −0.12 |
| Willingness to participate in tasks | Experimental | 31 | 2.81 ± 0.58 | 3.55 ± 0.52 | +0.74 ± 0.72 | 1.03 |
| Willingness to participate in tasks | Control | 31 | 2.97 ± 0.54 | 2.98 ± 0.56 | +0.01 ± 0.73 | 0.02 |
| Willingness to manage uncertainty | Experimental | 31 | 2.86 ± 0.42 | 3.52 ± 0.57 | +0.66 ± 0.65 | 1.01 |
| Willingness to manage uncertainty | Control | 31 | 2.96 ± 0.45 | 2.98 ± 0.65 | +0.02 ± 0.74 | 0.03 |

*Note. Reported descriptively only; not subjected to inferential testing owing to weaker subscale reliability. All inferential analyses used total scores.*
